# The prevalence and associated factors of moderate to severe depression symptoms among Fort McMurray residents during the COVID-19 Pandemic

**DOI:** 10.1192/j.eurpsy.2023.1672

**Published:** 2023-07-19

**Authors:** G. Obuobi-Donkor, E. Eboreime, R. Shalaby, B. Agyapong, E. Owusu, M. Adu, W. Mao, V. I. O. Agyapong

**Affiliations:** 1 Psychiatry, Dalhousie University, Halifax; 2 Psychiatry, University of Alberta, Edmonton, Canada

## Abstract

**Introduction:**

The Coronavirus disease (COVID-19) pandemic affected the mental health of many individuals, especially vulnerable communities, who have experienced multiple traumas.

**Objectives:**

To examine the prevalence and associated factors of likely major depressive disorder (MDD) among inhabitants of Fort McMurray.

**Methods:**

A study adopted a cross-sectional design, and questionnaires were distributed online. Sociodemographic, COVID-19-related, and clinical data were obtained. The Patient Health Questionnaire (PHQ-9) scale was used to assess likely MDD. SPSS version 25 used employed to analyze the data.

**Results:**

The prevalence of likely MDD among participants was 45%. Participants who desire mental health counselling are more likely to exhibit depression symptoms during the COVID-19 pandemic (OR = 5.48; 95% CI: 1.95–15.40). History of depression (OR = 4.64; 95% CI: 1.49–14.44) and hypnotics (OR = 5.72; 95% CI: 1.08–30.30) were more likely to experience depression symptoms during the pandemic than other participants without a history. Participants who received absolute support from the employer (OR = 3.50; 95% CI: 1.24–9.82) were protective against depression symptoms amid the pandemic.

**Conclusions:**

Clinical factors and employer support are associated with depression symptoms during the pandemic. Communities that have experienced multiple traumas need to reduce any psychopathology, and governmental bodies need to implement holistic policies to increase support to individuals during traumatic eras like the CVID-19 pandemic.

**Disclosure of Interest:**

None Declared

